# Glutaraldehyde-Crosslinked Bovine Serum Albumin Hydrogels for Efficient Cu^2+^, Ni^2+^, and Co^2+^ Removal from Water

**DOI:** 10.3390/polym18050633

**Published:** 2026-03-04

**Authors:** Dayana Lancheros-Ayala, Angie Méndez-Bautista, Giselle Barón-Gualdrón, Viviana Güiza-Argüello

**Affiliations:** Metallurgical Engineering and Materials Science Department, Universidad Industrial de Santander, Bucaramanga 680002, Colombia

**Keywords:** bovine serum albumin (BSA), heavy metal adsorption, protein-based hydrogels, water remediation, glutaraldehyde crosslinking

## Abstract

Heavy metal contamination remains a critical threat to water quality, particularly in effluents associated with industrial activities such as electroplating. This study presents an exploratory proof of concept for a simplified and low-requirement method to fabricate bovine serum albumin (BSA) hydrogels crosslinked with glutaraldehyde (GA) as protein-based adsorbents for Cu^2+^, Ni^2+^, and Co^2+^ removal. Hydrogel slabs were prepared using BSA concentrations of 20% and 25% (*w*/*v*) and GA in the 0.6–1.0% (*v*/*v*) range, with formulation adjustments guided by handling and aqueous stability. Swelling behavior was monitored for 23 days, and 0.9% (*v*/*v*) GA was selected to balance network expansion with hydrogel consistency. FT-IR confirmed preservation of protein functional groups in the crosslinked network, and TGA/DTG demonstrated multi-step thermal behavior consistent with hydrated protein matrices and a stabilizing effect of increased GA content. Metal removal tests at 50–100 ppm (Cu^2+^, Ni^2+^) and 70–100 ppm (Co^2+^) showed rapid removal approaching equilibrium within the first hours and improved performance at higher BSA content, achieving maximum removal percentages of 99.258% for Cu^2+^, 80.733% for Ni^2+^, and 76.070% for Co^2+^. Adsorption behaviors for Cu^2+^ and Co^2+^ aligned with the Langmuir model, while Ni^2+^ was better described by the Freundlich model. Although the scope is intentionally preliminary and limited to controlled synthetic systems, these results support GA-crosslinked BSA hydrogels as promising, easily fabricated adsorbents and establish a foundation for future studies on broader ion selectivity, competitive adsorption, and adsorption–desorption performance.

## 1. Introduction

By 2025, it is estimated that approximately half of the world’s population will face water-stress conditions [1]. UNESCO identifies heavy metal contamination as one of the factors exacerbating this crisis, undermining access to safe drinking water and degrading aquatic ecosystems. The situation becomes even more critical considering that only 27% of industrial wastewater and 58% of domestic wastewater are treated safely [1,2]. Moreover, many heavy metals pose a direct threat to human health due to their persistence, bioaccumulative behavior, and potential carcinogenicity, thereby constraining the safe use of water resources [3].

In recent years, increasing heavy-metal pollution originating primarily from agricultural, urban, and industrial sources has raised significant concern due to the deterioration of water quality [4,5]. Agriculture, through the recurrent use of fertilizers and pesticides, introduces metals such as arsenic (As), cadmium (Cd), and copper (Cu) into soils, which can percolate and ultimately contaminate groundwater. Likewise, leachates from landfills may contain metals such as lead (Pb) and zinc (Zn), while degradation of electronic waste, batteries, and coatings contributes to releases of other toxic metals, including mercury (Hg), nickel (Ni), and cobalt (Co) [6,7]. Industries such as metallurgy and mining discharge metallic ions during mineral extraction and refining processes, and the electroplating industry is a major contributor through acidic effluents generated in pickling and coating operations [8,9,10]. In countries such as China, the electroplating industry alone discharges approximately 400 million tons of wastewater annually [11,12]. Electroplating effluents can contain concentrations as high as 9069 ppm of copper [8], 87,755 ppm of nickel, and up to 2360 ppm of cobalt [13]. Among these, copper and nickel have been highlighted as critical contaminants in water sources impacted by mining, industrial, and agricultural activities [14,15].

Contamination by these metals has severe consequences for both human health and the environment. According to the U.S. Agency for Toxic Substances and Disease Registry, copper can cause gastrointestinal disorders, liver and kidney damage, anemia, and neurological impairments [16]. Cobalt exposure may lead to blood, liver, kidney, and heart damage [17], while nickel is classified as carcinogenic and prolonged exposure is associated with skin allergies, respiratory diseases, and increased risk of lung and nasal cancers [18,19]. In aquatic ecosystems, these metals can bioaccumulate, disrupting biodiversity and trophic chains [20].

Given this threat, a broad range of wastewater treatment technologies has been developed, including precipitation, coagulation/flocculation, membrane-based separations, adsorption, and advanced oxidation routes. Precipitation [21] remains widely used due to its simplicity and scalability, but efficiency can decline at trace concentrations. Coagulation [22] is extensively applied in large-scale plants, yet performance depends strongly on pH and reagent dosage and may generate secondary residues (e.g., sludge) requiring further management. Reverse osmosis [23] and membrane filtration [24] offer high removal efficiencies but often entail higher energy demand and operational costs, including fouling-related maintenance. In parallel, photocatalytic advanced oxidation processes are widely explored for the oxidative degradation of persistent organic contaminants; however, their translation beyond laboratory conditions is often constrained by reactor and light-delivery design, catalyst immobilization or recovery, and fouling/deactivation phenomena [25,26,27,28]. Microwave-enhanced catalytic/oxidation processes have also been proposed as process-intensification routes, leveraging rapid volumetric heating and microwave–material interactions to promote reactive species generation; nonetheless, practical deployment is frequently limited by reactor scale-up, energy-efficiency considerations, and the requirement for microwave-responsive catalysts and safe operating windows [29,30,31]. Importantly, while photocatalysis and microwave-assisted oxidation are typically aimed at degrading organic pollutants, dissolved heavy metals are not “decomposed” and thus generally require capture, separation, or immobilization strategies. For this reason, adsorption continues to attract strong interest for heavy-metal removal, particularly because it can be operationally simple, modular, and adaptable through rational material selection, with the additional possibility of regeneration and resource recovery when feasible [32].

Conventional adsorbents such as activated carbon have been widely used due to their large surface area and versatility. However, performance may decline in environments with relatively low concentrations of metal ions, and regeneration can be energy-intensive. Consequently, a wide range of bio-derived adsorbents has been investigated, including cellulose, alginate, chitosan, starch, and protein-based materials (e.g., sericin, casein, and spirulina). These biopolymers are biodegradable and often present functional groups such as –OH, –NH_2_, or –COOH capable of coordinating metal ions, yet they may also show limited wet-strength, sensitivity to solution chemistry, and variable selectivity under multi-ion conditions [7,33]. Recent hydrogel-based biosorbents (including chemically crosslinked systems) continue to demonstrate the promise of biopolymer networks for heavy-metal capture while highlighting the need for reproducible fabrication and adequate performance in aqueous environments [34].

In this context, bovine serum albumin (BSA) emerges as a promising material for heavy metal decontamination. This abundant plasma protein exhibits a high density of metal-binding functionalities (–SH, –NH_2_, =NH, and –COOH), enabling chelation and transport of diverse metal ions [35,36,37,38], and offering a broader interaction landscape than many polysaccharide-based biopolymers [39,40]. Importantly, this broad interaction capability is valuable for broad-spectrum remediation of mixed-metal streams; however, high selectivity (i.e., strong preference for one ion over others) is typically more difficult to achieve and must be quantified under competitive conditions. Recent work has explored BSA-based architectures including hydrogels, aerogels, membranes, and microbubbles, in some cases achieving high removal efficiencies (up to 99%) and demonstrating the versatility of albumin-derived networks [41,42,43,44,45]. At the same time, many reported routes rely on comparatively complex or multi-step fabrication approaches, underscoring the need for alternative methodologies that prioritize simplicity and reproducibility for scale-relevant development.

This work presents an accessible and rapid route to fabricate bovine serum albumin (BSA) hydrogels crosslinked with glutaraldehyde (GA) as protein-based adsorbents for Cu^2+^, Ni^2+^, and Co^2+^ removal. The novelty of this study lies not only in employing a bio-derived polymeric network for metal removal, but also in the deliberately simplified, low-requirement fabrication strategy that yields stable hydrogel slabs with fast gelation through straightforward processing steps. By systematically narrowing the GA window to balance swelling with hydrogel consistency and by comparing two practically relevant BSA contents, this study provides an exploratory yet reproducible foundation for protein-hydrogel adsorbents tailored to water remediation. The results establish a clear formulation baseline and performance benchmarks that can guide future optimization toward mixed-ion systems, regeneration, and real effluents.

In contrast to prior BSA-based adsorbents that often rely on more complex or multi-step fabrication routes, the present work advances a streamlined proof-of-concept approach to generate GA-crosslinked BSA hydrogels using simple, handling-guided formulation adjustments. By correlating aqueous stability, swelling behavior, and ion-specific adsorption performance within a narrow but instructive design space, we offer an accessible framework for developing protein-derived hydrogel adsorbents for Cu^2+^, Ni^2+^, and Co^2+^ capture. Importantly, the novelty here is not framed as optimized industrial readiness, but as the establishment of a low-barrier experimental platform that is sufficiently robust to motivate expanded studies of selectivity, competitive adsorption (which are particularly important because true selectivity is challenging and competition can alter apparent capacities), and reuse-oriented performance.

## 2. Materials and Methods

### 2.1. Materials

Bovine serum albumin (BSA; Fraction V, fatty acid-free; Sigma-Aldrich, St. Louis, MO, USA) and glutaraldehyde (GA; pentane-1,5-dial; Sigma-Aldrich, St. Louis, MO, USA) were used to prepare the hydrogel precursor solutions. The GA was supplied as a 25% aqueous solution (Sigma-Aldrich). Aqueous solutions of the studied metal ions were prepared using copper(II) sulfate anhydrous (Supelco, Bellefonte, PA, USA), nickel(II) sulfate hexahydrate (Supelco), and cobalt(II) sulfate heptahydrate (Sigma-Aldrich, St. Louis, MO, USA). All solutions were prepared with deionized water (DI H_2_O, resistivity 18.2 MΩ·cm).

### 2.2. Design of the Assembly for Fabricating BSA Hydrogels

**Figure 1:**
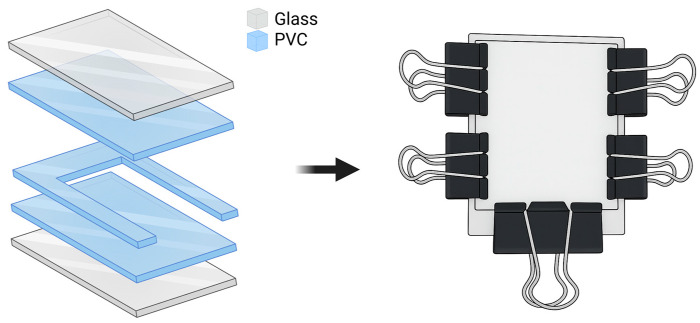
Design of the assembly for the fabrication of BSA hydrogel slabs. Created in BioRender. Ayala, D. (2025) https://BioRender.com/63p4ndr (accessed on 6 December 2025).

To control hydrogel geometry, an assembly was designed for the fabrication of BSA hydrogel slabs. A rectangular mold was constructed from a flexible poly(vinyl chloride) (PVC) sheet. An internal U-shaped PVC insert served as a spacer to minimize hydrogel adhesion and facilitate demolding. Two glass plates were used as external walls to provide stability to the mold, and all components were secured using double clip-type clamps, as illustrated in Figure 1.

### 2.3. Preparation of BSA Precursor Solutions

BSA precursor solutions were prepared in DI water (18.2 MΩ·cm) at concentrations selected to yield final hydrogel BSA contents of 20% and 25% (*w*/*v*) after mixing with the corresponding GA working solution (Section 2.4). Immediately before use, the BSA precursor solutions were centrifuged at 6000 rpm to remove entrapped air bubbles.

### 2.4. BSA Hydrogel Fabrication

Hydrogels were fabricated by mixing the BSA precursor solutions (Section 2.3) with aqueous GA working solutions (prepared from the 25% stock solution) at a 1.5:1 (*v*/*v*) ratio (BSA solution:GA solution). GA was evaluated at three final levels in the hydrogel precursor solution—0.6, 0.9, and 1.0% (*v*/*v*)—to examine the effect of crosslinker dose on the physical properties of the resulting hydrogels. Here, GA (% *v*/*v*) is reported as the final volume fraction of GA (added from the GA working solution, which was prepared by diluting the 25% stock) relative to the total hydrogel precursor volume after mixing.

**Figure 2:**
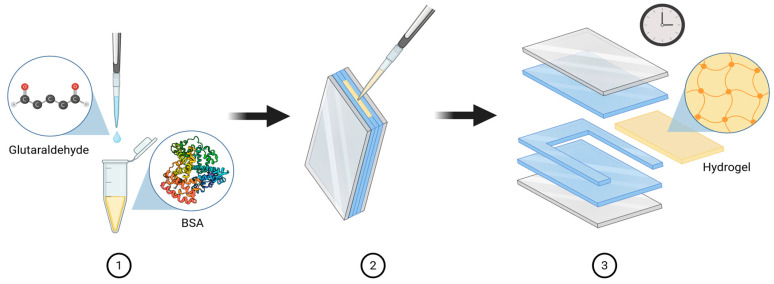
BSA hydrogel fabrication process. Created in BioRender. Ayala, D. (2025) https://BioRender.com/63p4ndr (accessed on 6 December 2025).

As shown in Figure 2, the GA working solution was added to the BSA precursor solution and mixed by pipetting until visually homogeneous. The mixture was poured into the preassembled mold and allowed to gel for 8 min at ambient temperature, after which the hydrogel slabs were demolded.

Buffer and pH conditions: all precursor solutions were prepared in DI water and gels were cast without buffer and without intentional pH adjustment; therefore, gelation proceeded at the native pH of the precursor mixture (approximately pH 5.5–6.5), which was measured using pH strips (immediately after homogenization, prior to casting).

### 2.5. Swelling and Stability Tests in Aqueous Media

**Figure 3:**
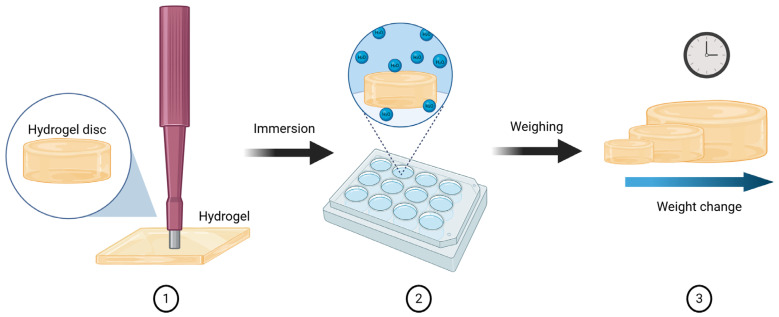
Evaluation of the swelling behavior and aqueous stability of the fabricated hydrogels. in BioRender. Ayala, D. (2025) https://BioRender.com/63p4ndr (accessed on 6 December 2025).

To evaluate the stability of the BSA hydrogels in an aqueous environment, disk-shaped samples were prepared using a 10 mm punch cutter (Figure 3). For each sample, both the initial mass and dimensions were recorded before transferring the hydrogel into a 12-well plate containing 4 mL of deionized water per well. From this point onward, the mass and dimensions of the samples were monitored over a total period of 23 days to quantify the percentage of mass change over time associated with swelling and hydrolysis processes, thereby assessing the stability of the hydrogels in an aqueous medium.

The corresponding mass change was estimated using the following equation [46]:

(1) $$\%\;C_{m}=\frac{m_{f}\;-m_{0}}{m_{0}}\times 100\;$$

where *% C_m_* represents the percentage of mass change of the hydrogel, *m*_0_ is the initial weight of the hydrogel, and *m_f_* is the weight of the hydrogel at time *t*.

### 2.6. Fourier Transform Infrared Spectroscopy (FT-IR)

Fourier transform infrared (FT-IR) spectroscopy was used to identify the functional chemical groups present in the samples. Spectra were collected using an FT-IR spectrometer (Cary 630 FT-IR, Agilent Technologies, Santa Clara, CA, USA) equipped with an attenuated total reflectance (ATR) accessory, over the wavenumber range of 4000–650 cm^−1^, with a spectral resolution of 2 cm^−1^ [47].

### 2.7. Thermogravimetric Analysis (TGA)

The thermal stability of the hydrogels was evaluated by thermogravimetric analysis (TGA) using a thermogravimetric analyzer (TGA 5500, TA Instruments, New Castle, DE, USA). Samples were heated from 30 to 800 °C under a nitrogen atmosphere at a heating rate of 10 °C min^−1^. The mass of each hydrogel sample was continuously recorded as a function of temperature, and the corresponding thermogravimetric (TG) curves were obtained [48].

### 2.8. Speciation Diagrams

Speciation diagrams for Cu^2+^, Ni^2+^, and Co^2+^ were generated using the Hydra-Medusa software v0.1.1 to analyze the distribution of species as a function of pH for ionic concentrations ranging from 5 to 100 ppm. Based on these diagrams and the isoelectric point of BSA (pH = 5.1–5.5), an initial pH of 5.0 was selected for all ion removal experiments. The solutions were prepared without buffer; pH was adjusted to 5.0 at the start using dilute HNO_3_ and verified with a calibrated pH meter. During the adsorption tests, pH was not actively controlled (no buffer) and was not continuously monitored. Because the experiments were designed as a low-requirement screening study, pH drift during ion removal was not tracked; future work will quantify and control pH during adsorption.

### 2.9. Metal Removal Tests

**Figure 4:**
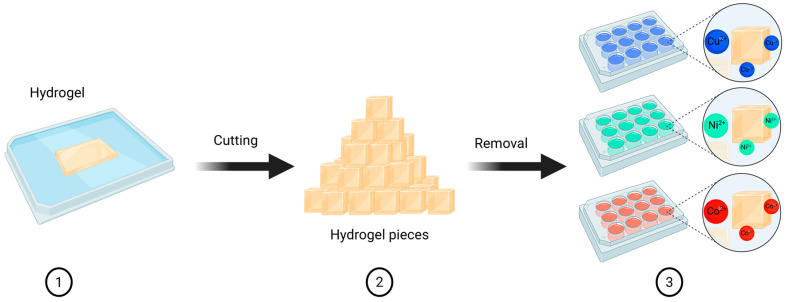
Preparation of adsorption tests for Cu^2+^, Ni^2+^, and Co^2+^ ions in synthetic aqueous solutions. Created in BioRender. Ayala, D. (2025) https://BioRender.com/63p4ndr (accessed on 6 December 2025).

In the metal ion removal tests for Cu^2+^, Ni^2+^, and Co^2+^, the hydrogels were allowed to swell in DI water at room temperature prior to use. The swollen hydrogels were then cut into approximately 2 × 2 mm square pieces. Subsequently, between 900 and 1000 mg of hydrogel per well were transferred to a plate containing 3.5 mL of the target metal ion solution per well (Figure 4). The variables studied were removal time (3, 15, and 24 h) and ionic concentration of the medium (50 and 100 ppm). These contact times were selected as practical screening points to assess equilibration and removal performance; the limited number and spacing of time points were not intended to support detailed kinetic modeling.

Based on the collected data, the percentage of Cu^2+^, Ni^2+^, and Co^2+^ removed was determined by atomic absorption spectroscopy using a Thermo Electron S4 SOLAAR (Cambridge, UK) instrument and the following equation [49]:

(2) $$\%\;R=\frac{C_{0}\;-C_{f}}{C_{0}}\times 100$$

where *% R* is the percentage of metal removed (the fraction of dissolved metal removed from solution in batch tests), and *C*_0_ and *C_f_* represent the initial and final metal concentrations in the test solution (mg/L), respectively.

### 2.10. Adsorption Isotherms

For these experiments, the samples were prepared following the same procedure as in the metal removal tests. Once the hydrogel pieces were obtained, metal ion removal was carried out in Cu^2+^, Ni^2+^, and Co^2+^ solutions at concentrations of 5, 15, and 50 ppm for 5 h (the estimated time to reach adsorption equilibrium). The adsorption capacity of the BSA hydrogels was determined by atomic absorption spectroscopy (Thermo Electron S4 SOLAAR) using Equation (3), as described below [49]:

(3) $$q=\frac{\left(C_{0}\;-C_{f}\right)}{m}\times V\;$$

where *q* is the adsorption capacity (mg ion/g hydrogel, the mass of metal adsorbed per unit mass of hydrogel), *V* is the volume of the synthetic solution containing the metal contaminant (L), and *m* is the mass of the hydrogel used [49]. The data were subsequently analyzed using the Langmuir and Freundlich isotherm models. Towards this, the nonlinear form of each model was applied, as shown below. For the Langmuir model [49]:

(4) $$q_{e}=\frac{q_{m}\;K_{L}\;C_{e}}{1\;+\;K_{L}\;C_{e}}$$

where *q_m_* is the maximum adsorption capacity of the adsorbent, *C_e_* is the equilibrium concentration after 5 h of removal (mg/L), *q_e_* is the adsorption capacity of the hydrogel at equilibrium, and *K_L_* is the Langmuir constant, representing the affinity between the adsorbate and the adsorbent (L/mg). It is important to note here that *q*, *q_e_*, and *q_m_* are mass-normalized adsorption capacity descriptors, distinct from % R. In the case of the Freundlich model [49]:

(5) $$q_{e}=K_{F}\;C_{e}^{\frac{1}{n}}$$

where *K_F_* and *n* are Freundlich constants that define the affinity of the adsorbate for the adsorbent (L/mg) and the adsorption intensity, respectively.

## 3. Results and Discussion

### 3.1. Preparation of BSA Hydrogels

**Figure 5:**
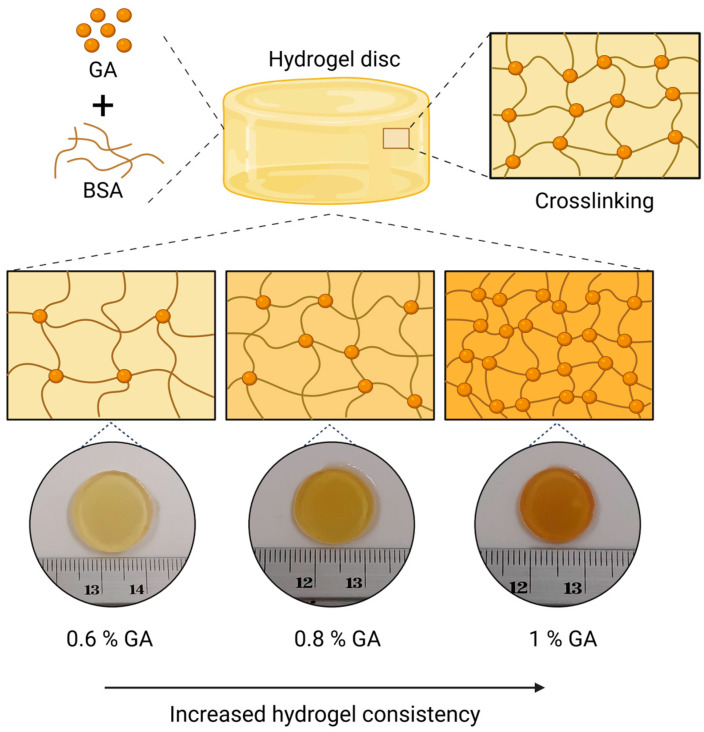
Influence of GA concentration on the physical properties of BSA hydrogels (24 h after immersion in water). Created in BioRender. Ayala, D. (2025) https://BioRender.com/63p4ndr (accessed on 6 December 2025).

When hydrogels were prepared with final concentrations of 20 and 25% (*w*/*v*) BSA crosslinked with different concentrations of GA, a progressive change in both color and consistency was observed as the GA percentage increased (Figure 5). Hydrogels containing 0.6% (*v*/*v*) GA were difficult to handle due to their soft consistency, likely resulting from a low degree of crosslinking (see Supplementary Figure S1). Therefore, this formulation was excluded from subsequent experiments, and the study range was adjusted to 0.8%, 0.9%, and 1.0% (*v*/*v*) GA for the swelling and stability tests.

### 3.2. Swelling and Stability Tests in Aqueous Medium

Hydrogel swelling behavior provides not only information about the amount of fluid a material can absorb but also critical insights for designing, optimizing, and predicting its performance in applications such as the removal of heavy metals from wastewater [46]. Swelling behavior is influenced by hydrophilic functionality, the degree of crosslinking, and the resulting hydrated network free volume and diffusion pathways available for water and solute transport [50]. A higher swelling ratio indicates a more expanded hydrated network, which may increase the accessibility of potential binding sites and facilitate ion transport within the hydrogel matrix, thereby potentially enhancing adsorption [46].

**Figure 6:**
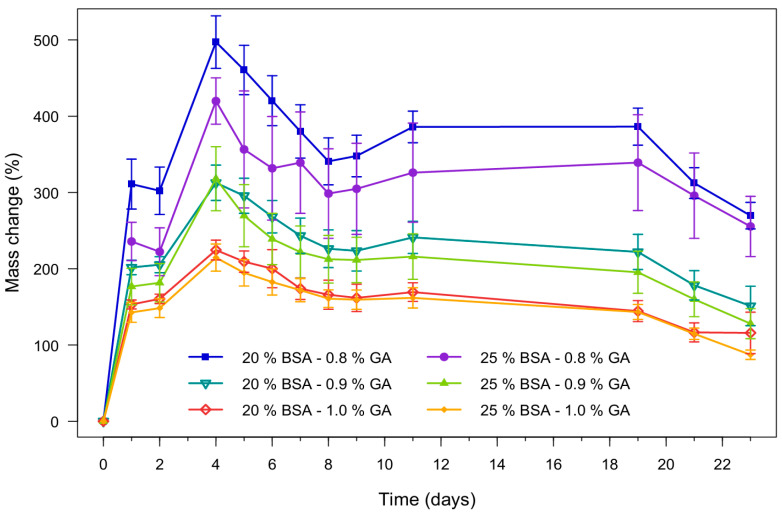
Stability in aqueous medium of BSA hydrogels prepared using different protein concentrations and crosslinker levels.

However, excessive expansion may reduce structural stability, increasing degradability and potentially limiting practical reuse. Conversely, a very low swelling ratio may reduce accessibility to adsorption sites, hindering the penetration of metal ions into adsorption sites within the hydrogel. Nevertheless, increasing the degree of crosslinking typically enhances the structural stability of hydrogel adsorbents and improves practical handling during aqueous exposure. Based on the above considerations, swelling and stability tests in aqueous medium were performed on hydrogels containing 20 and 25% (*w*/*v*) BSA and 0.8%, 0.9%, and 1.0% (*v*/*v*) GA (n = 6) over a period of 23 days. The results are presented in Figure 6.

When analyzing the data, although no negative mass changes were found, the decreases observed at certain points could indicate the onset of degradation and the consequent mass loss of the hydrogel. On the other hand, a consistent pattern was identified: as the crosslinker concentration (% *v*/*v* GA) increases, the mass change decreases. Similarly, it was observed that for each set of samples with the same GA percentage, those containing 25% (*w*/*v*) BSA exhibited a lower degree of swelling compared to those with lower concentration of protein. According to the literature, this behavior is attributed to the protein content within the hydrogel, since a higher protein concentration reduces the available space within the network for water uptake, thereby promoting interactions between polymer chains rather than with water molecules (see Figure 5). Therefore, 0.9% (*v*/*v*) GA was selected for subsequent experiments in order to obtain hydrogels capable of expanding their polymeric network to favor adsorption without compromising hydrogel consistency, thereby supporting handleability and stability that are desirable for practical handling and aqueous stability.

### 3.3. Fourier Transform Infrared Spectroscopy (FT-IR)

**Figure 7:**
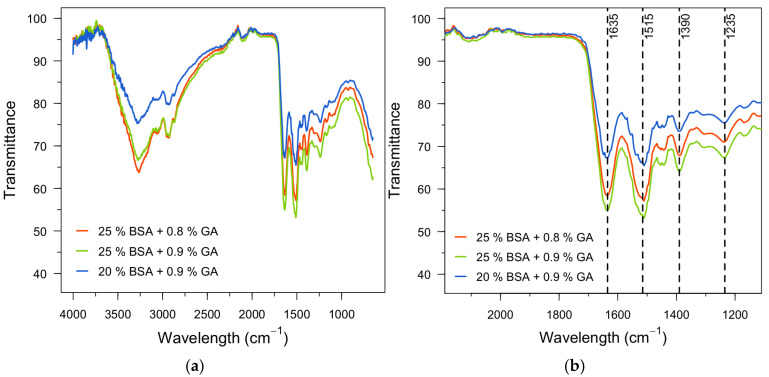
FT-IR spectra of glutaraldehyde-crosslinked BSA hydrogels, with different GA concentration (0.8 and 0.9%) and BSA content (20 and 25% *w*/*v*).

Structural characterization was conducted through FT-IR to: (i) confirm that the fabricated materials retain the characteristic chemical functionalities of protein-based networks, and (ii) evaluate whether changes in GA dose and BSA content produce detectable spectral differences consistent with chemical crosslinking (Figure 7). Across all evaluated formulations, the spectra display the expected bands of albumin/protein materials: a broad band centered at ~3275 cm^−1^ assigned to overlapping O–H/N–H stretching vibrations, and aliphatic C–H stretching bands around ~2900 cm^−1^ (Figure 7a,b) [44,51].

The amide bands that define the protein backbone are clearly observed for every hydrogel formulation, indicating preservation of the polypeptide framework after gelation and aqueous handling. Specifically, the dominant amide I band appears at ~1635 cm^−1^, while amide II is present around ~1515 cm^−1^, and amide III around ~1235 cm^−1^ (Figure 7b) [51,52]. A particularly relevant feature for the adsorption function of these hydrogels is the persistence of the band near ~1390 cm^−1^, associated with carboxylate groups (–COO^−^). The retention of this signal across the tested GA/BSA conditions suggests that a substantial fraction of carboxylate functionalities remains available after crosslinking, supporting the premise that the network conserves O-donor binding sites that can participate in divalent metal uptake [42,47].

Regarding the effect of GA concentration (25% BSA with 0.8 vs. 0.9% GA; Figure 7a), the overall spectral profile remains highly similar, which is consistent with crosslinking that modifies a subset of reactive amine sites without altering the fundamental protein backbone signatures. In GA-crosslinked protein systems, GA typically reacts with nucleophilic amino groups (e.g., ε-NH_2_ of lysine and N-termini), producing a covalently connected network often described as involving Schiff-base-type (imine) linkages or related GA-derived structures. However, in FT-IR the imine-region assignment is intrinsically challenging here because potential C=N contributions overlap with the amide I envelope (peptide C=O), making unambiguous identification by FT-IR alone difficult. Consequently, while the FT-IR spectra are fully consistent with successful formation of a crosslinked protein network, confirmation of specific imine bonding would require complementary surface/chemical-state analysis (e.g., XPS) [53,54].

Nonetheless, the obtained FT-IR spectra provide a structural baseline: the hydrogels retain canonical protein functional groups relevant to adsorption and show spectra consistent with GA-crosslinked protein networks.

### 3.4. Thermogravimetric Analysis

**Figure 8:**
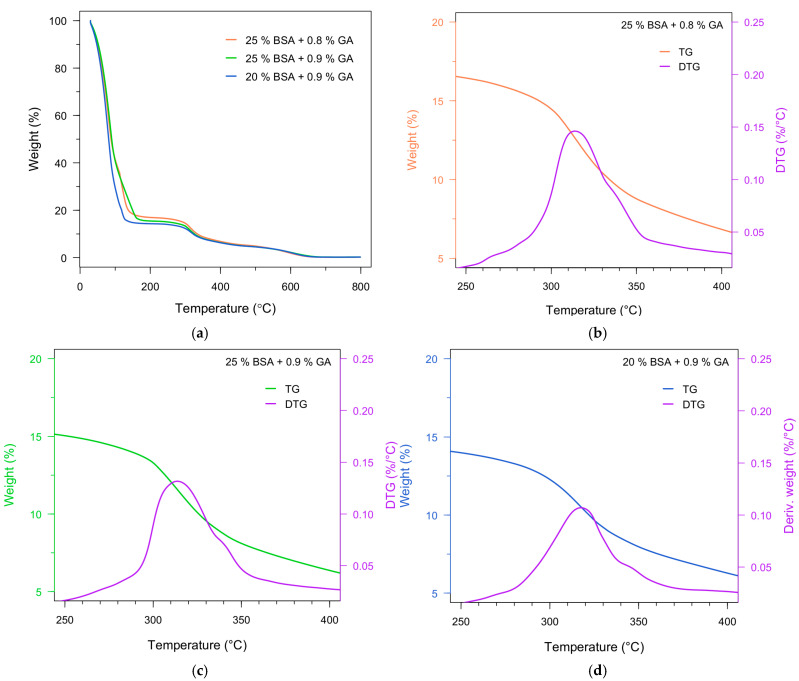
Thermogravimetric analysis of fresh BSA hydrogels crosslinked with different glutaraldehyde concentrations and BSA contents: (**a**) Comparative TG curves; (**b**) 25% BSA + 0.8% GA; (**c**) 25% BSA + 0.9% GA; (**d**) 20% BSA + 0.9% GA.

Thermogravimetric analysis (TGA) and derivative thermogravimetry (DTG) were employed to compare the thermal stability and multi-step degradation behavior of fresh (hydrated) BSA–GA hydrogels as a function of GA concentration and BSA content (Figure 8). Because the samples were analyzed in their hydrated state, the TG/DTG profiles capture two coupled contributions: (i) low-temperature mass loss dominated by removal of free and weakly bound water retained within the hydrogel network, and (ii) higher-temperature decomposition of the crosslinked protein matrix. This multi-step degradation profile is characteristic of highly hydrated polymer networks and protein-based hydrogels [53,55,56].

All formulations exhibit an initial mass-loss stage below ~150 °C associated primarily with evaporation of free or loosely bound water (Figure 8a) [55]. Notably, the formulation with lower solids fraction (20% BSA at 0.9% GA) shows a more pronounced early mass loss, consistent with a higher relative water content or a less dense network that retains a larger fraction of free (less strongly bound) water [54]. This interpretation is quantitatively supported by the *T*_50_ parameter (temperature at 50% mass loss) reported in Table 1: *T*_50_ decreases markedly from 90.125 °C (25% BSA + 0.9% GA) to 81.53 °C (20% BSA + 0.9% GA). In hydrated samples, *T*_50_ is strongly influenced by the water fraction; therefore, the lower *T*_50_ at 20% BSA reflects earlier attainment of 50% total mass loss due to higher water-driven mass loss at relatively low temperatures [47,56].

The second degradation region (approximately 200–350 °C) corresponds to the main thermal decomposition of the BSA–GA network, involving cleavage of peptide bonds, disruption of protein secondary structure, and decomposition of crosslink-related covalent structures (Figure 8b–d) [51,57]. Within the 25% BSA series, increasing GA from 0.8 to 0.9% produces modest but consistent increases in standard thermal-stability descriptors: *T_onset_* increases from 286.272 to 287.721 °C, and *T_max_* increases from 308.958 to 312.472 °C (Table 1). These shifts, although not large, align with the expected effect of a slightly higher crosslink density—namely, restricting chain mobility and delaying the onset and peak rate of backbone decomposition by requiring higher thermal energy to activate bond scission and network collapse pathways.

Above ~350 °C, a third stage becomes evident and is generally attributed to decomposition/oxidation of carbonaceous residues and breakdown of more thermally stable fragments generated during earlier steps [47,51]. While the present comparison focuses on formulation contrasts (GA dose and BSA content), the persistence of a multi-step profile across all samples reinforces that the materials behave as crosslinked protein networks rather than as simple physical gels.

At constant GA content (0.9%), reducing the BSA concentration from 25% to 20% produces two coupled effects that should be interpreted together in the context of hydrated hydrogel thermograms. First, the marked decrease in *T*_50_ from 90.125 °C (25% BSA + 0.9% GA) to 81.53 °C (20% BSA + 0.9% GA) indicates that the lower-BSA formulation contains a higher relative water fraction and exhibits weaker water binding within the network, thereby reaching 50% total mass loss earlier during the low-temperature water-removal regime (Table 1). Second, despite a slightly lower decomposition onset (*T_onset_* = 282.309 °C for 20% BSA vs. 287.721 °C for 25% BSA), the DTG peak temperature (*T_max_*) shifts upward (324.063 °C at 20% BSA vs. 312.472 °C at 25% BSA).

In hydrated protein networks, this combination can arise when a less concentrated protein matrix begins to undergo structural disruption earlier, yet reaches the maximum rate of decomposition at a higher temperature due to differences in heat and mass transport through the matrix, water-induced plasticization effects, or a broader distribution of crosslinked versus weakly crosslinked domains. From a practical standpoint, these trends emphasize that BSA content strongly governs the apparent thermal response because it controls the network solids fraction and water retention, which in turn modulate both the early mass-loss behavior and the kinetics of the principal decomposition event. Overall, the TGA/DTG results directly demonstrate that increasing GA content (from 0.8 to 0.9%) yields a measurable, directionally consistent improvement in onset/peak decomposition temperatures at fixed BSA, whereas changing BSA content (from 25% to 20% at fixed GA) produces a larger shift in low-temperature mass loss (water retention) and significantly alters *T*_50_, highlighting solids fraction/network density as a dominant lever in the thermal behavior of these hydrated BSA–GA hydrogels.

### 3.5. Metal Removal Tests

**Figure 9:**
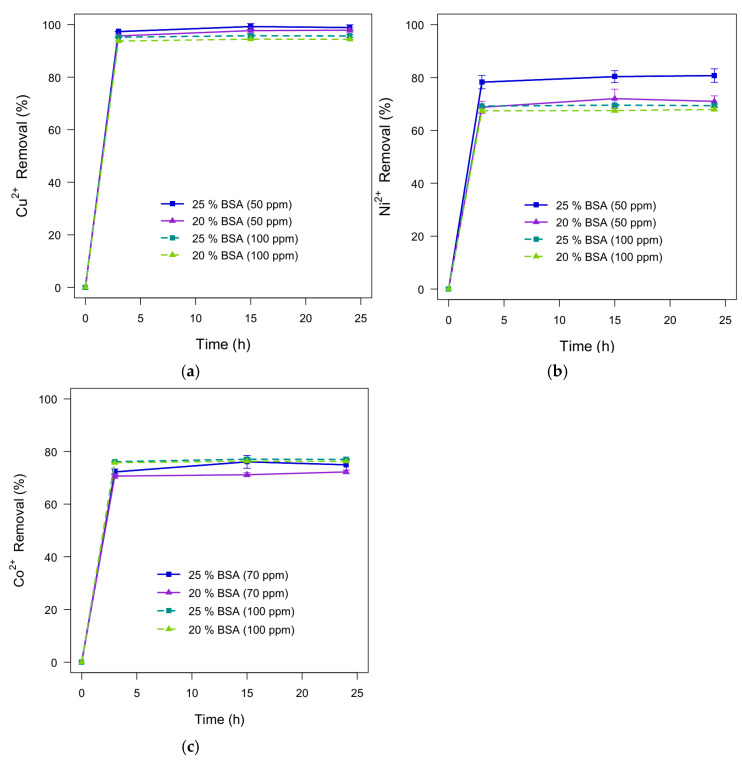
Effect of BSA concentration (20% and 25% *w*/*v*) on the metal ion removal percentage of the fabricated hydrogels: (**a**) Cu^2+^; (**b**) Ni^2+^; (**c**) Co^2+^.

The previously selected formulations composed of 20% and 25% (*w*/*v*) BSA crosslinked with 0.9% (*v*/*v*) glutaraldehyde were used to evaluate the influence of BSA concentration on the performance of the hydrogels in metal removal percentage (% R). After allowing the hydrogels to swell in water for three days, removal tests were conducted in triplicate (n = 3) independently for each synthetically contaminated solution. The metal concentrations employed were 50 and 100 ppm for Cu^2+^ and Ni^2+^ solutions, and 70 and 100 ppm for the Co^2+^ solution. In this way, the effect of hydrogel contact time (3, 15, and 24 h) under high and low concentration conditions was analyzed. Removal tests were carried out at pH 5.0, which was selected based on Hydra–Medusa speciation diagrams and the BSA isoelectric point. The results (see Figure 9) show that removal at 3 h was already close to the values observed at 15 and 24 h in most cases, suggesting a rapid approach toward equilibrium under the investigated conditions. When compared with similar systems, the rapid approach toward equilibrium is consistent with a hydrated network that enables efficient transport and access to adsorption sites under the investigated conditions.

Most of the analyzed samples exhibited a decrease in removal percentage as the concentration of the test solutions increased. This widely reported trend is commonly interpreted as reflecting faster saturation of available adsorption sites at higher metal concentrations. On the other hand, it was observed that hydrogels with higher BSA content showed an increase in removal percentage, as presented in Table 2. This trend is consistent with the higher BSA content providing a greater density of potential adsorption sites within the hydrogel network. Additionally, the hydrogels demonstrated a marked affinity for Cu^2+^, which was the most strongly adsorbed ion among the configurations analyzed.

Based on these results, hydrogels containing 25% (*w*/*v*) BSA and 0.9% (*v*/*v*) GA exhibited higher performance compared to those containing 20% (*w*/*v*) BSA and 0.9% (*v*/*v*) GA, reaching removal values of up to 99% in the case of Cu^2+^. Furthermore, the study of contact time revealed that metal removal is a rapid process, with near-equilibrium behavior observed within the first three hours under the conditions tested. Nevertheless, longer contact times were assessed to confirm stability of removal performance.

### 3.6. Adsorption Isotherms

The study of adsorption isotherms is useful for interpreting adsorbate–adsorbent interactions under equilibrium conditions and for obtaining comparative descriptors of adsorption behavior within a defined concentration window [58,59]. In this work, the Langmuir and Freundlich nonlinear models were applied because they offer complementary descriptions of adsorption on idealized homogeneous versus heterogeneous surfaces and are widely used as first-pass frameworks for comparing adsorption trends across systems [60,61]. The Langmuir model assumes monolayer adsorption on a homogeneous surface with a finite number of equivalent sites, whereas the Freundlich model represents adsorption on heterogeneous surfaces with a distribution of site energies and the possibility of multilayer adsorption [61,62,63]. Here, isotherm experiments were conducted using initial metal-ion concentrations of 5, 15, 25, and 50 ppm to provide an exploratory equilibrium benchmark for the selected formulation.

**Figure 10:**
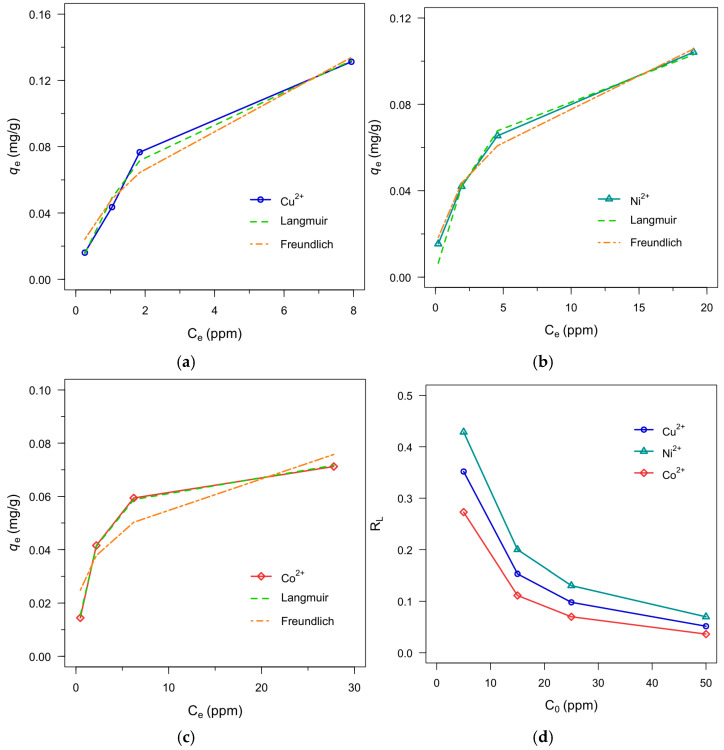
Nonlinear Langmuir and Freundlich model descriptions of the experimental equilibrium data for GA-crosslinked BSA hydrogels (25% (*w*/*v*) BSA, 0.9% (*v*/*v*) GA) within the tested initial concentration range (5–50 ppm). (**a**) Cu^2+^; (**b**) Ni^2+^; (**c**) Co^2+^. (**d**) Separation factor (*R_L_*) values for all ions (Cu^2+^, Ni^2+^, and Co^2+^), indicating adsorption favorability (0 < *R_L_* < 1) across 5–50 ppm.

Figure 10 summarizes the isotherm analysis for hydrogels composed of 25% (*w*/*v*) BSA and 0.9% (*v*/*v*) GA, using triplicate measurements (n = 3) at each initial concentration. Within the tested concentration range, the regression coefficients (R^2^; Table 3) indicate that Ni^2+^ showed closer alignment with the Freundlich model (Figure 10b), while Cu^2+^ and Co^2+^ were better described by the Langmuir model (Figure 10a,c). These differences should be interpreted as preliminary descriptors of ion-dependent adsorption behavior rather than definitive evidence of a single mechanism, particularly given the limited number of concentration points used in this proof-of-concept screening. Nonetheless, the observed model preferences are consistent with the expectation that a protein-based hydrogel presents a chemically diverse adsorption landscape in which site heterogeneity and solution chemistry can contribute to ion-dependent behavior and mixed adsorption characteristics [60]. In addition, the calculated separation factor (*R_L_*) values (Figure 10d) fall within the favorable adsorption range (0 < *R_L_* < 1) for all ions across 5–50 ppm, supporting that adsorption proceeds favorably under the conditions evaluated.

Within the Freundlich framework, the fitted parameters (n > 1) indicate favorable adsorption and the *K_F_* values provide a comparative descriptor of adsorption capacity within the tested range. Consistent with the assumptions of the Freundlich model, the Ni^2+^ data may reflect adsorption on a heterogeneous set of sites and possible multilayer-type behavior; however, these interpretations remain model-based and are not direct evidence of layer structure or specific binding-site energetics. In contrast, the parameters derived from the Langmuir adsorption isotherm model were consistent with the removal trends, and the calculated *R_L_* values (0 < *R_L_* < 1) support favorable adsorption under the conditions evaluated (Figure 10d). However, upon comparing the data, it was observed that although Cu^2+^ displayed a lower affinity constant (*K_L_*) with the hydrogel, it exhibited the highest adsorption capacity (*q_m_*). This behavior, previously reported in the literature, suggests that, even if the average interaction strength represented by *K_L_* is not the highest, the system can exhibit a higher overall uptake capacity (*q_m_*) within the tested range, potentially reflecting a higher density of adsorption sites and/or greater accessibility within the hydrated network [60].

**Figure 11:**
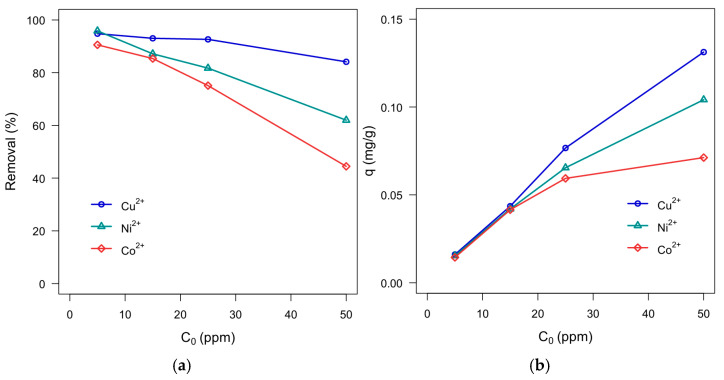
Comparison of hydrogel adsorption performance across all three metal ions. (**a**) Removal percentage at concentrations ranging from 5 to 50 ppm. (**b**) Adsorption capacity per gram of hydrogel.

Moreover, Figure 11 compares hydrogel adsorption performance across all three metal ions and shows consistently higher removal and adsorption capacity for Cu^2+^ under the investigated conditions. This trend is qualitatively consistent with widely reported Cu^2+^ coordination behavior toward N- and O-donor ligands (e.g., amino and carboxylate functionalities), in protein-based systems. Some reports further discuss the possible influence of Cu^2+^ Jahn–Teller distortion in stabilizing certain coordination geometries [62,64,65,66,67,68]; however, the present study does not directly probe coordination environments or functional-group-specific binding. Accordingly, the above considerations are provided only as mechanistic context, and definitive attribution would require targeted spectroscopic and/or computational validation (e.g., FT-IR/XPS and DFT) in future work.

**Figure 12:**
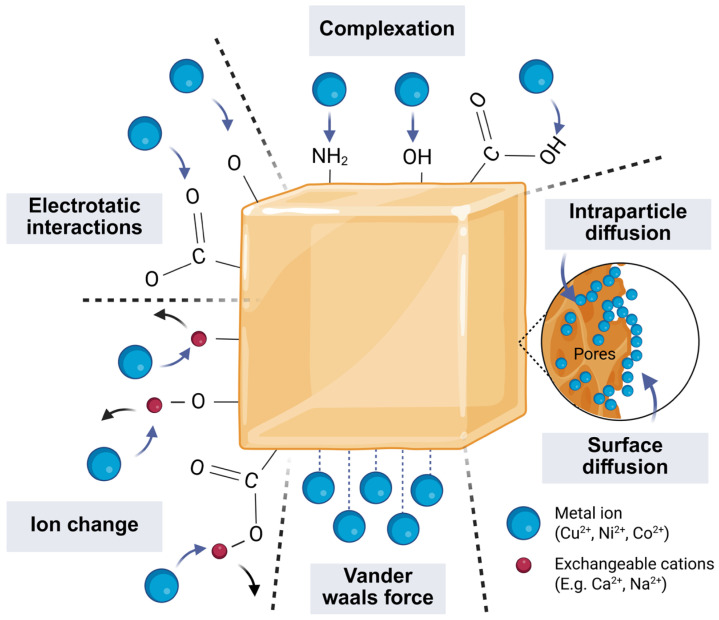
Conceptual schematic of proposed interaction pathways between GA-crosslinked BSA hydrogels and divalent metal ions (e.g., coordination/complexation, electrostatic contributions, and non-specific interactions). The schematic is intended as a hypothesis-generating framework; definitive assignment requires spectroscopic confirmation (e.g., FT-IR/XPS). Created in BioRender. Ayala, D. (2025) https://BioRender.com/63p4ndr (accessed on 6 December 2025).

Furthermore, Figure 12 summarizes plausible interaction pathways that are compatible with BSA chemistry and the observed adsorption trends, including complexation, electrostatic contributions, ion exchange, and non-specific interactions. These assignments are presented as mechanistic hypotheses; FT-IR supports the presence of adsorption-relevant functional groups in the network, but identifying the active binding moieties and metal–ligand coordination environment will require complementary analyses such as XPS, which are reserved for future work.

## 4. Conclusions

This study demonstrates a simple and rapid approach to fabricate glutaraldehyde (GA)-crosslinked bovine serum albumin (BSA) hydrogels as protein-based adsorbents for Cu^2+^, Ni^2+^, and Co^2+^ removal from aqueous media. By screening formulation handleability and aqueous stability and then analyzing swelling behavior, an intermediate GA content (0.9% *v*/*v*) was identified as a practical compromise between network expansion and adequate hydrogel consistency. Within the tested formulation range, higher BSA content improved metal removal performance, with particularly strong removal percentage of Cu^2+^.

FT-IR spectra confirmed that the hydrogels retain the canonical functional groups of protein-based networks after gelation and aqueous handling (amide I/II/III bands), and that key adsorption-relevant functionalities such as carboxylate groups remain detectable across the tested formulations, consistent with preservation of O- and N-donor binding sites in the crosslinked matrix. Moreover, thermogravimetric analysis of hydrated hydrogels revealed the expected multi-step mass-loss behavior, comprising low-temperature water removal followed by decomposition of the crosslinked protein matrix at higher temperatures. Within the 25% BSA series, increasing GA from 0.8% to 0.9% produced a directionally consistent increase in thermal-stability descriptors, supporting the expected stabilizing role of crosslink density. In contrast, decreasing BSA content at fixed 0.9% GA produced a larger shift in the low-temperature regime (T_50_), consistent with higher relative water fraction and altered water retention in the less concentrated network.

The optimized hydrogel formulation (25% (*w*/*v*) BSA and 0.9% (*v*/*v*) GA) displayed substantial swelling capacity (up to ~ 300% mass increase) and supported adequate handling and feasibility for preliminary remediation assessment in synthetic systems. Equilibrium analysis further highlighted ion-dependent adsorption behavior, with Cu^2+^ and Co^2+^ aligning with the Langmuir model and Ni^2+^ better described by the Freundlich model, suggesting that distinct interaction modes may operate within the protein network.

Recent evidence indicates that hydrogel adsorption performance is closely tied to structural features such as hydrated network accessibility, transport pathways, and active-site availability [46]. In this context, the combined swelling and adsorption results obtained here suggest that the synthesized BSA hydrogels form a flexible hydrated network with an adequate density of accessible functional groups, capable of promoting efficient interaction with Cu^2+^, Ni^2+^, and Co^2+^ under the investigated conditions.

Given the intentionally exploratory scope, these findings should be interpreted as establishing feasibility rather than defining ultimate performance limits. Nonetheless, the combination of accessible fabrication, rapid gelation, aqueous stability, and promising removal efficiencies supports GA-crosslinked BSA hydrogels as low-complexity, bio-derived adsorbents and provides a clear formulation baseline for further development (see Table 4).

As summarized in Table 4, the $q_{m}$ values obtained for the present GA–BSA hydrogels (0.077–0.177 mg/g) are lower than those reported for other BSA-containing adsorbents that were evaluated and reported on a dry-mass basis. In contrast, in this proof-of-concept study, the adsorption capacity was calculated per gram of hydrated hydrogel (i.e., wet mass after pre-swelling), which yields lower mass-normalized capacities because the majority of the sample mass corresponds to water rather than sorbent solids. Consequently, direct numerical comparisons of *q_m_*_across studies should be interpreted cautiously unless the mass basis (dry vs. wet), adsorbent dose, and concentration window are explicitly aligned. Importantly, adsorption capacity alone is not a sufficient metric to judge practical utility, particularly for applications at low-to-moderate dissolved metal concentrations, where rapid removal, operational simplicity, and ease of solid–liquid separation can be equally decisive. Under the investigated conditions, the GA–BSA hydrogels achieved high removal fractions (up to ∼99% for Cu^2+^), approached equilibrium rapidly, and were produced as macroscopic, easily handled slabs within minutes (Table 4), enabling straightforward physical recovery without additional filtration steps. Consistent with recent discussions of hydrogel-based adsorbents [50], these attributes motivate evaluating performance in a multi-metric manner (capacity together with kinetics, stability, and practical recoverability), and they support the present system as a promising low-complexity platform for further optimization.

**Limitations and Outlook**

This work is presented as an exploratory proof of concept and therefore carries several limitations. First, adsorption experiments were conducted using single-metal synthetic solutions; consequently, competitive adsorption and selectivity under mixed-ion conditions remain to be established. Second, although swelling behavior was monitored over an extended period, adsorption–desorption cycling and regeneration were not investigated; thus, long-term reusability and capacity retention across cycles remain unknown. Establishing reusability (via desorption efficiency, capacity retention over multiple cycles, and preservation of hydrogel integrity) will be a priority in future work to determine whether these BSA–GA hydrogels are better suited for single-use deployment or multi-cycle operation. Third, while single-ion testing enabled baseline comparisons across formulations, performance in multicomponent systems may differ due to competitive adsorption and ion-speciation effects; therefore, evaluating mixed Cu^2+^/Ni^2+^/Co^2+^ solutions will be an important next step to determine preferential binding and behavior under more realistic conditions. Moreover, although removal efficiency was evaluated as a function of contact time, the experimental design included a limited number and spacing of time points (3, 15, and 24 h), which do not support robust kinetic fitting; accordingly, kinetic models such as pseudo-first-order or pseudo-second-order were not applied in this study. Finally, the study focused on a narrow but practically motivated formulation window of BSA and GA contents to prioritize fabrication simplicity and handleability, which may not capture the full performance envelope achievable through broader compositional or processing optimization.

Despite these constraints, the results provide a useful and reproducible baseline for advancing protein-based hydrogel adsorbents. Future studies should examine the influence of gelation time on network architecture, expand affinity screening to additional environmentally relevant ions (e.g., Zn^2+^, Cd^2+^, Pb^2+^), evaluate competitive adsorption in multicomponent solutions representative of electroplating- or mining-related wastewater, incorporate expanded time-course sampling to enable kinetic modeling, and develop regeneration strategies compatible with protein networks. Together, these efforts will help define the practical boundaries, scalability, and application readiness of this low-barrier BSA hydrogel platform for water treatment.

## Figures and Tables

**Table 1:** Thermogravimetric parameters of BSA–GA hydrogels.

Composition	*T_onset_*	*T_max_*	*T*_50_
25% BSA + 0.8% GA	286.272	308.958	88.285
25% BSA + 0.9% GA	287.721	312.472	90.125
20% BSA + 0.9% GA	282.309	324.063	81.53

**Table 2:** Ion removal performance of BSA hydrogels at varying metal ion concentrations.

Metal Ion	Concentration (ppm)	*% R_max_* ^1^	
		25% BSA	20% BSA
Cu^2+^	50	99.258	97.865
	100	95.758	94.462
Ni^2+^	50	80.733	72.015
	100	69.559	67.931
Co^2+^	70	76.070	72.232
	100	77.090	76.324

^1^ *% R_max_* represents the maximum removal percentage recorded during the experimental time period analyzed.

**Table 3:** Nonlinear Langmuir and Freundlich isotherm parameters for the metal adsorption of BSA hydrogels.

Metal Ion	Langmuir Isotherm Model				Freundlich Isotherm Model		
	*q_m_* (mg/g)	*K_L_* (L/mg)	*R_L_* ^1^	R^2^	*K_F_* (L/mg)	*n* (L/mg)	R^2^
Cu^2+^	0.177	0.368	0.051–0.352	0.992	0.047	1.996	0.966
Ni^2+^	0.123	0.267	0.070–0.429	0.980	0.034	2.581	0.992
Co^2+^	0.077	0.533	0.036–0.273	0.999	0.030	3.648	0.877

^1^ The values reported for *R_L_* correspond to the range obtained for samples at the highest (50 ppm) and lowest (5 ppm) initial ion concentrations, respectively.

**Table 4:** Comparison of previously reported BSA-containing materials used for metal ion adsorption applications.

Reference	Material	Fabrication Time	Target Ion	C_0_ (ppm)	Removal Percentage, % R	*q_m_* (mg/g)	Contact Time
[39], 2025	Thermally induced BSA hydrogel	15 h	Cd^2+^	2.54	98.54	-	24 h
			Pb^2+^	2.13	97.99		
[42], 2022	BSA amyloid fibril aerogel	>140 h	Cu^2+^	25–250	80	82.2	1.5 h
[53], 2021	3D-printed GO-PDA-BSA aerogel	>50 h	Cr (VI)	25–200	93	45.05	96 h
			Pb^2+^		94	43.76	
[43], 2018	PA–BSA TFC membrane (forward osmosis)	1 h	Cu^2+^	2000	>99	-	-
[45], 2014	BSA-coated microbubbles	~2 h	Cu^2+^		98	-	1.5 h
			Ni^2+^	10–100	16		
			Co^2+^		13		
**This work**	**GA-crosslinked BSA hydrogels**	**8 min**	**Cu^2+^**	**50–100**	**99.258**	**0.177**	**5 h**
			**Ni^2+^**		**80.733**	**0.123**	
			**Co^2+^**		**76.070**	**0.077**	

**Note:** In this work, $q_{m}\;$ is expressed per gram of hydrated (wet) hydrogel after pre-swelling. Reported $q_{m}\;$ values in the literature ([36,54]) are based on dry adsorbent mass; therefore, direct numerical comparison should be interpreted cautiously.

## Data Availability

Data available in a publicly accessible repository: The original data presented in the study are openly available in Mendeley Data at https://data.mendeley.com/datasets/6tws94jjzm/2 (accessed on 6 December 2025).

## Supplementary Materials

The following supporting information can be downloaded at: https://www.mdpi.com/article/10.3390/polym18050633/s1, A. Supplementary Figure S1. Preparation of BSA hydrogels. Representative photographs of a BSA hydrogel slab and hydrogel disks prepared with final BSA concentrations of 20 and 25% (*w*/*v*), crosslinked with different glutaraldehyde (GA) contents, after 1, 3, and 9 days of immersion in deionized water. Increasing GA content led to a progressive change in both color and consistency; samples prepared with 0.6% (*v*/*v*) GA were difficult to handle due to their soft consistency, likely reflecting a low crosslinking density. Over time, lower-GA hydrogels exhibited more pronounced dimensional changes (greater swelling), whereas higher-GA formulations remained comparatively stable, consistent with the stability trends shown in Figure 6. As discussed in Section 3.2, this reduced swelling at higher GA content is attributed to a denser, more rigid crosslinked network that restricts water uptake. B. R code for nonlinear regression analysis of isotherm data.

## References

[1] Unesco (2024). The United Nations World Water Development Report 2024: Water for Prosperity and Peace; Executive Summary.

[2] Health O.W., Habitat U.N. (2024). Progress on the Proportion of Domestic and Industrial Wastewater Flows Safely Treated–Mid-Term Status of SDG Indicator 6.3.1 and Acceleration Needs, with a Special Focus on Climate Change, Wastewater Reuse and Health.

[3] Alengebawy A., Abdelkhalek S.T., Qureshi S.R., Wang M.-Q. (2021). Heavy Metals and Pesticides Toxicity in Agricultural Soil and Plants: Ecological Risks and Human Health Implications. Toxics.

[4] Zhang J., Hao Z., Liu X., Wang B., Guo W., Yan J. (2024). Surface Water Quality Evaluation and Pollution Source Analysis at the Confluence of the Wei River and Yellow River, China. Water.

[5] Fadlillah L.N., Utami S., Rachmawati A.A., Jayanto G.D., Widyastuti M. (2023). Ecological risk and source identifications of heavy metals contamination in the water and surface sediments from anthropogenic impacts of urban river, Indonesia. Heliyon.

[6] Boethling R., Fenner K., Howard P., Klečka G., Madsen T., Snape J.R., Whelan M.J. (2009). Environmental Persistence of Organic Pollutants: Guidance for Development and Review of POP Risk Profiles. Integr. Environ. Assess. Manag..

[7] Rahman M.L., Fui C.J., Ting T.X., Sarjadi M.S., Arshad S.E., Musta B. (2020). Polymer Ligands Derived from Jute Fiber for Heavy Metal Removal from Electroplating Wastewater. Polymers.

[8] Gu J.-n., Liang J., Chen C., Li K., Zhou W., Jia J., Sun T. (2020). Treatment of real deplating wastewater through an environmental friendly precipitation-electrodeposition-oxidation process: Recovery of silver and copper and reuse of wastewater. Sep. Purif. Technol..

[9] Zhou Y., Liu Z., Bo A., Tana T., Liu X., Zhao F., Sarina S., Jia M., Yang C., Gu Y. (2020). Simultaneous removal of cationic and anionic heavy metal contaminants from electroplating effluent by hydrotalcite adsorbent with disulfide (S2-) intercalation. J. Hazard. Mater..

[10] Moersidik S.S., Nugroho R., Handayani M., Kamilawati, Pratama M.A. (2020). Optimization and reaction kinetics on the removal of Nickel and COD from wastewater from electroplating industry using Electrocoagulation and Advanced Oxidation Processes. Heliyon.

[11] Xiong W., Yang M., Wang J., Wang H., Zhao P., Li Z., Liu B., Kong X., Duan H., Zhao Y. (2023). Removal, recycle and reutilization of multiple heavy metal ions from electroplating wastewater using super-stable mineralizer Ca-based layered double hydroxides. Chem. Eng. Sci..

[12] Gong W.-J., Niu Z.-F., Wang X.-R., Zhao H.-P. (2021). How the Soil Microbial Communities and Activities Respond to Long-Term Heavy Metal Contamination in Electroplating Contaminated Site. Microorganisms.

[13] Cai X., Zheng X., Zhang D., Iqbal W., Liu C., Yang B., Zhao X., Lu X., Mao Y. (2019). Microbial characterization of heavy metal resistant bacterial strains isolated from an electroplating wastewater treatment plant. Ecotoxicol. Environ. Saf..

[14] Marrugo-Negrete J., Pinedo-Hernández J., Marrugo-Madrid S., Díez S. (2021). Assessment of trace element pollution and ecological risks in a river basin impacted by mining in Colombia. Environ. Sci. Pollut. Res..

[15] Torrance K.W., Redwood S.D., Cecchi A. (2021). The impact of artisanal gold mining, ore processing and mineralization on water quality in Marmato, Colombia. Environ. Geochem. Health.

[16] Agency for Toxic Substances and Disease Registry (2024). Toxicological Profile for Copper.

[17] Agency for Toxic Substances and Disease Registry (2004). Toxicological Profile for Cobalt.

[18] Agency for Toxic Substances and Disease Registry (2024). Toxicological Profile for Nickel.

[19] International Agency for Research on Cancer (2004). Tobacco Smoke and Involuntary Smoking.

[20] Argumedo C.D. (2021). Bioconcentración de metales pesados (Zn, Hg, Pb) en tejidos de Ariopsis felis y Diplodus annularis en el río Ranchería, Norte de Colombia. Rev. La Fac. Med. Vet. Y Zootec..

[21] Xu W., Yang H., Mao Q., Luo L., Deng Y. (2022). Removal of Heavy Metals from Acid Mine Drainage by Red Mud–Based Geopolymer Pervious Concrete: Batch and Long–Term Column Studies. Polymers.

[22] Banch T.J.H., Hanafiah M.M., Alkarkhi A.F.M., Abu Amr S.S. (2019). Factorial Design and Optimization of Landfill Leachate Treatment Using Tannin-Based Natural Coagulant. Polymers.

[23] Ji K., Liu C., He H., Mao X., Wei L., Wang H., Zhang M., Shen Y., Sun R., Zhou F. (2023). Research Progress of Water Treatment Technology Based on Nanofiber Membranes. Polymers.

[24] Kolya H., Kang C.-W. (2023). Next-Generation Water Treatment: Exploring the Potential of Biopolymer-Based Nanocomposites in Adsorption and Membrane Filtration. Polymers.

[25] Hübner U., Spahr S., Lutze H., Wieland A., Rüting S., Gernjak W., Wenk J. (2024). Advanced oxidation processes for water and wastewater treatment–Guidance for systematic future research. Heliyon.

[26] Iyyappan J., Gaddala B., Gnanasekaran R., Gopinath M., Yuvaraj D., Kumar V. (2024). Critical review on wastewater treatment using photo catalytic advanced oxidation process: Role of photocatalytic materials, reactor design and kinetics. Case Stud. Chem. Environ. Eng..

[27] Horváth E., Gabathuler J., Bourdiec G., Vidal-Revel E., Benthem Muñiz M., Gaal M., Grandjean D., Breider F., Rossi L., Sienkiewicz A. (2022). Solar water purification with photocatalytic nanocomposite filter based on TiO2 nanowires and carbon nanotubes. npj Clean Water.

[28] Yang J., Guo S., Dong H., Liu Y., Wang J., Quan G., Zhang X., Lei J., Liu N. (2025). Enhanced visible-light-driven photocatalysis of ibuprofen by NH2 modified MIL-53(Fe) graphene aerogel: Performance, mechanism, pathway and toxicity assessment. Colloids Surf. A Physicochem. Eng. Asp..

[29] Xia H., Li C., Yang G., Shi Z., Jin C., He W., Xu J., Li G. (2022). A review of microwave-assisted advanced oxidation processes for wastewater treatment. Chemosphere.

[30] Lee J., Weon S., Lee S.S.S., Yun E.-t., Chung M.W., Kim C., Wang H., Fortner J.D. (2025). Microwave-enhanced catalytic degradation of organic compounds with silica-coated iron oxide nanocrystals via fenton-like reaction pathway. npj Clean Water.

[31] Tang C., Lin B., Niu H., Zheng K., Liu Y., Chen X., Zhong K., Zhu R., Chen Y., Li H. (2025). Highly dispersed ZIF-67 derived cobalt nanoparticle supported on g-C3N4 for rapid degradation of sulfamethoxazole by Fenton-like oxidation: Enhanced adsorption and electron transfer. J. Colloid Interface Sci..

[32] Sheraz M., Sun X.-F., Siddiqui A., Hu S., Song Z. (2025). Research Advances in Natural Polymers for Environmental Remediation. Polymers.

[33] Ayach J., El Malti W., Duma L., Lalevée J., Al Ajami M., Hamad H., Hijazi A. (2024). Comparing Conventional and Advanced Approaches for Heavy Metal Removal in Wastewater Treatment: An In-Depth Review Emphasizing Filter-Based Strategies. Polymers.

[34] Taha A.M., Mustafa F.H.A., Ibrahim H.E., Mohamadein L.I., Anwar Z.M., Elsharaawy R.F.M. (2025). Adsorptive removal of heavy metal ions from wastewater using shrimp chitosan-cysteine-glutaraldehyde hydrogel as a sustainable biosorbent. Int. J. Biol. Macromol..

[35] Flora S.J.S., Pachauri V. (2010). Chelation in Metal Intoxication. Int. J. Environ. Res. Public Health.

[36] Saraswathi M.S.A., Mahalakshmi S., Vetrivel S., Divya K., Rana D., Nagendran A. (2018). Separation of bovine serum albumin and humic acid contaminants from aqueous stream using tailored poly (amide imide) ultrafiltration membranes. J. Environ. Chem. Eng..

[37] Lu J., Stewart A.J., Sadler P.J., Pinheiro T.J., Blindauer C.A. (2008). Albumin as a zinc carrier: Properties of its high-affinity zinc-binding site. Biochem. Soc. Trans..

[38] Moriya M., Ho Y.-H., Grana A., Nguyen L., Alvarez A., Jamil R., Ackland M.L., Michalczyk A., Hamer P., Ramos D. (2008). Copper is taken up efficiently from albumin and α2-macroglobulin by cultured human cells by more than one mechanism. Am. J. Physiol.-Cell Physiol..

[39] Mondal A., Haque M., Aggarwal A., Kalita M., Singha Roy A. (2025). Protein-based hydrogel for environmental remediation: Removal of hazardous metal ions and toxic organic dyes from wastewater. J. Mol. Liq..

[40] Yang F., Yang Q., Chen M., Luo C., Chen W., Yang P. (2021). Toxic metal ion sequestration by amyloid-mediated fast coacervation. Cell Rep. Phys. Sci..

[41] Upadhyay A., Narula A., Rao C.P. (2020). Copper-Based Metallogel of Bovine Serum Albumin and Its Derived Hybrid Biomaterials as Aerogel and Sheet: Comparative Study of the Adsorption and Reduction of Dyes and Nitroaromatics. ACS Appl. Bio Mater..

[42] Wei N., Yang J., Dong K., Fang Y., Qin Z. (2022). Amino-functionalized bovine serum albumin amyloid fibrils aerogel for absorbing copper from water. J. Clean. Prod..

[43] Zhao X., Liu C. (2018). Efficient removal of heavy metal ions based on the optimized dissolution-diffusion-flow forward osmosis process. Chem. Eng. J..

[44] Nazari A.M., Cox P.W., Waters K.E. (2015). Biosorptive flotation of copper ions from dilute solution using BSA-coated bubbles. Miner. Eng..

[45] Nazari A.M., Cox P.W., Waters K.E. (2014). Biosorption of copper, nickel and cobalt ions from dilute solutions using BSA-coated air bubbles. J. Water Process Eng..

[46] Sandu T., Chiriac A.-L., Zaharia A., Iordache T.-V., Sarbu A. (2025). New Trends in Preparation and Use of Hydrogels for Water Treatment. Gels.

[47] Lee D.H., Ahn H.J., Lee J., Woo H.C. (2025). Biodegradable PVA–Alginate Bio-Based Polymers Incorporating Cardanol-Based Polyols for Antibacterial Applications. Polymers.

[48] Aswathy S.H., NarendraKumar U., Manjubala I. (2022). Physicochemical Properties of Cellulose-Based Hydrogel for Biomedical Applications. Polymers.

[49] Torres-Badajoz S.G., Rodríguez-Núñez J.R., López-Ramírez E., Peña-Caballero V., Villa-Lerma A.G., Madera-Santana T.J., Rodríguez-Carrillo M.G., Castillo O.S. (2022). Kinetic and Equilibrium Studies of Cr(VI) Adsorption Using Glutaraldehyde-Crosslinked Chitosan Beads. Iran. J. Sci. Technol. Trans. A Sci..

[50] Darban Z., Shahabuddin S., Gaur R., Ahmad I., Sridewi N. (2022). Hydrogel-Based Adsorbent Material for the Effective Removal of Heavy Metals from Wastewater: A Comprehensive Review. Gels.

[51] Zainuddin A.N., Razif R., Nizam A.A., Maarof M., Fadilah N.I., Kim Y.-H., Mahmoudi E., Fauzi M.B. (2025). Kelulut Honey-Incorporated Hybrid Gelatin-PVA Hydrogel for Wound Healing: Fabrication and In Vitro Characterization. Polymers.

[52] Sun M., Wang Y., Yao L., Li Y., Weng Y., Qiu D. (2022). Fabrication and Characterization of Gelatin/Polyvinyl Alcohol Composite Scaffold. Polymers.

[53] Masud A., Zhou C., Aich N. (2021). Emerging investigator series: 3D printed graphene-biopolymer aerogels for water contaminant removal: A proof of concept. Environ. Sci. Nano.

[54] Esfahan Z.M., Izhar S., Ismail M.H.S., Yoshida H. (2021). Subcritical water treatment of bovine serum albumin pathway to produce superabsorbent biomaterial as green technology. Mater. Today Sustain..

[55] Bekbayeva L., Mun G.A., Yermukhambetova B.B., Negim E.-S., Irmukhametova G., Al Azzam K.M., Nechipurenko S.V., Efremov S.A., Yermaganbetov M., Samy M. (2025). Synthesis and Characterization of Biodegradable Polymer Blends Based on Chitosan. Polymers.

[56] Li X., Gao H., Wang Q., Liu S. (2024). Enhancing the Toughness of PAA/LCNF/SA Hydrogel through Double-Network Crosslinking for Strain Sensor Application. Polymers.

[57] Mahmoudi C., Tahraoui Douma N., Mahmoudi H., Iurciuc C.E., Popa M., Hamcerencu M., Andrițoiu C.V. (2024). Developing and Characterizing a Biocompatible Hydrogel Obtained by Cross-Linking Gelatin with Oxidized Sodium Alginate for Potential Biomedical Applications. Polymers.

[58] Khan M., Shafi M., Raza J., Ahmed I.A., Zada A., Narasimharao K., Sun X. (2025). Mechanistic breakthroughs in affordable adsorbents for heavy metal remediation: An in-depth exploration of next-generation sustainable water purification technologies. J. Hazard. Mater. Adv..

[59] Mnasri-Ghnimi S., Frini-Srasra N. (2019). Removal of heavy metals from aqueous solutions by adsorption using single and mixed pillared clays. Appl. Clay Sci..

[60] de Castro-Alves L., Yáñez-Vilar S., González-Goméz M.A., Garcia-Acevedo P., Arnosa-Prieto Á., Piñeiro-Redondo Y., Rivas J. (2024). Understanding adsorption mechanisms and metal ion selectivity of superparamagnetic beads with mesoporous CMK-3 carbon and commercial activated carbon. Microporous Mesoporous Mater..

[61] Mohammed A.H., Shartooh S.M., Trigui M. (2025). Biosorption and Isotherm Modeling of Heavy Metals Using Phragmites australis. Sustainability.

[62] Pogorilyi R.P., Pylypchuk I., Melnyk I.V., Zub Y.L., Seisenbaeva G.A., Kessler V.G. (2017). Sol-Gel Derived Adsorbents with Enzymatic and Complexonate Functions for Complex Water Remediation. Nanomaterials.

[63] Tenea A.-G., Dinu C., Rus P.A., Ionescu I.A., Gheorghe S., Iancu V.I., Vasile G.G., Pascu L.F., Chiriac F.L. (2024). Exploring adsorption dynamics of heavy metals onto varied commercial microplastic substrates: Isothermal models and kinetics analysis. Heliyon.

[64] Yildiz U., Kemik Ö.F., Hazer B. (2010). The removal of heavy metal ions from aqueous solutions by novel pH-sensitive hydrogels. J. Hazard. Mater..

[65] Lü R., Cao Z., Shen G. (2008). Comparative study on interaction between copper (II) and chitin/chitosan by density functional calculation. J. Mol. Struct. THEOCHEM.

[66] Li M., Zhang L., Wang M., Meng X., Shao P., Yang L., Zhao C., Cheng N., Wang H. (2023). A nanofiber with a p-π conjugated structure designed based on the Jahn-Teller effect for the removal of cupric tartrate from wastewater. J. Colloid Interface Sci..

[67] Gao D.L., Lin W.W., Lin Q.J., Dai F.F., Xue Y.X., Chen J.H., Liu Y.X., Huang Y., Yang Q. (2023). Remarkable adsorption capacity of Cu2+-doped ZnAl layered double hydroxides and the calcined materials toward phosphate. J. Environ. Chem. Eng..

[68] Pestov A., Bratskaya S. (2016). Chitosan and Its Derivatives as Highly Efficient Polymer Ligands. Molecules.

