# Supplementary material for: Glutaraldehyde-Crosslinked Bovine Serum Albumin Hydrogels for Efficient Cu2+, Ni2+, and Co2+ Removal from Water
Source: Polymers (Basel). 2026 Mar 4;18(5):633. doi: 10.3390/polym18050633 (PMC12987242; doi:10.3390/polym18050633)
Supplement: Supplementary file 1 [file polymers-18-00633-s001.zip › polymers-4059944-supplementary.pdf]

## Supplementary Information

### A. Supplementary Figure S1.

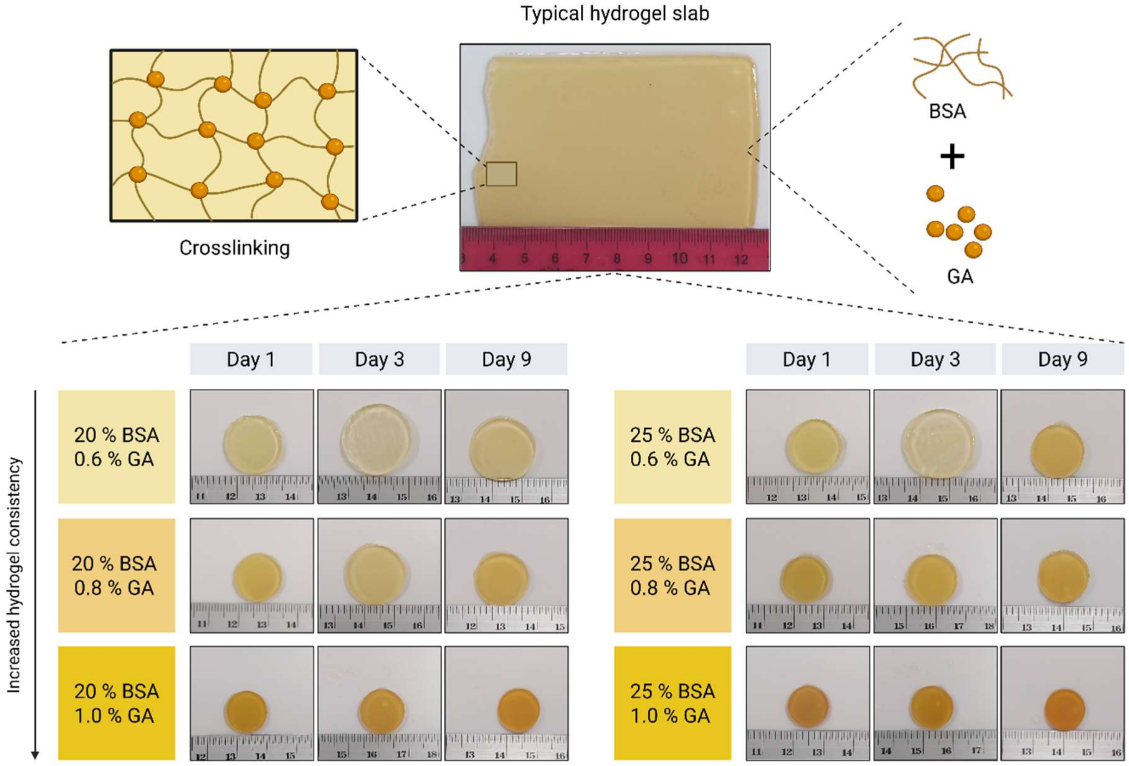


**Supplementary Figure S1.** Preparation of BSA hydrogels. Representative photographs of a BSA hydrogel slab and hydrogel disks prepared with final BSA concentrations of 20 and 25% (w/v), crosslinked with different glutaraldehyde (GA) contents, after 1, 3, and 9 days of immersion in deionized water. Increasing GA content led to a progressive change in both color and consistency; samples prepared with 0.6% (v/v) GA were difficult to handle due to their soft consistency, likely reflecting a low crosslinking density. Over time, lower-GA hydrogels exhibited more pronounced dimensional changes (greater swelling), whereas higher-GA formulations remained comparatively stable, consistent with the stability trends shown in Figure 6. As discussed in Section 3.2, this reduced swelling at higher GA content is attributed to a denser, more rigid crosslinked network that restricts water uptake.

B. **R code for nonlinear regression analysis of isotherm data.**

```r
# =============================
# NONLINEAR REGRESSION
# LANGMUIR AND FREUNDLICH ISOTHERMS + R_L
# BASE R
# =============================


library(readxl)
file.choose()
datos <- read_excel("C:\\Users\\Betty\\Desktop\\Isotherm Data for Non-Linear Regression.xlsx")


# =============================
# FITTING FUNCTIONS
# =============================

# Nonlinear Langmuir isotherm
ajuste_langmuir <- function(Ce, qe) {
  modelo <- nls(
    qe ~ (qm * KL * Ce) / (1 + KL * Ce),
    start = list(qm = max(qe), KL = 1),
    control = nls.control(maxiter = 200, warnOnly = TRUE)
  )
  pred <- predict(modelo)
  R2 <- 1 - sum((qe - pred)^2) / sum((qe - mean(qe))^2)
  list(coef = coef(modelo), R2 = R2)
}

# Nonlinear Freundlich isotherm
ajuste_freundlich <- function(Ce, qe) {
  modelo <- nls(
    qe ~ KF * Ce^(1/n),
    start = list(KF = 1, n = 2),
    control = nls.control(maxiter = 200, warnOnly = TRUE)
  )
  pred <- predict(modelo)
  R2 <- 1 - sum((qe - pred)^2) / sum((qe - mean(qe))^2)
  list(coef = coef(modelo), R2 = R2)
}

# =============================
# MODEL FITTING BY METAL
# =============================


# CU2+
L_Cu <- ajuste_langmuir(datos$CECU, datos$PCU)
F_Cu <- ajuste_freundlich(datos$CECU, datos$PCU)


# NI2+
L_Ni <- ajuste_langmuir(datos$CENI, datos$PNI)
F_Ni <- ajuste_freundlich(datos$CENI, datos$PNI)


# CO2+
L_Co <- ajuste_langmuir(datos$CECO, datos$PCO)
F_Co <- ajuste_freundlich(datos$CECO, datos$PCO)

# =============================
# RESULTS
# =============================
```

```r
cat("\nLANGMUIR - Cu2+\n"); print(L_Cu)
cat("\nFREUNDLICH - Cu2+\n"); print(F_Cu)


cat("\nLANGMUIR - Ni2+\n"); print(L_Ni)
cat("\nFREUNDLICH - Ni2+\n"); print(F_Ni)


cat("\nLANGMUIR - Co2+\n"); print(L_Co)
cat("\nFREUNDLICH - Co2+\n"); print(F_Co)



# =============================
# SEPARATION FACTOR RL
# =============================


# Initial concentrations
C0 <- datos$C


# Extract KL
KL_Cu <- L_Cu$coef["KL"]
KL_Ni <- L_Ni$coef["KL"]
KL_Co <- L_Co$coef["KL"]


# Calculate RL
RL <- data.frame(
  C_mg_L = C0,
  RL_Cu = 1 / (1 + KL_Cu * C0),
  RL_Ni = 1 / (1 + KL_Ni * C0),
  RL_Co = 1 / (1 + KL_Co * C0)
)


cat("\nSEPARATION FACTOR RL\n")
print(RL)
```
